# Longitudinal Monitoring of Plasma Circulating Tumour DNA Enables the Prediction of Early Relapse in Patients with Non-Hodgkin Lymphoma: A Case Series

**DOI:** 10.3390/diagnostics11112055

**Published:** 2021-11-05

**Authors:** Hongyan Ji, Xiaolu Long, Jia Gu, Jin Jin, Xia Mao, Zhiqiong Wang, Heng Ma, Liting Chen

**Affiliations:** 1 Department of Haematology, Tongji Hospital, Tongji Medical College, Huazhong University of Science and Technology, Wuhan 430030, China; jihongyan1111@163.com (H.J.); longxiaolu@tjh.tjmu.edu.cn (X.L.); meilixinqing1936@163.com (J.G.); jin820@hust.edu.cn (J.J.); maoxia2009@163.com (X.M.); wangzq_427@163.com (Z.W.); 2 FNA Cytology Examining Room, Tongji Hospital, Tongji Medical College, Huazhong University of Science and Technology, Wuhan 430030, China; maheng609485@163.com

**Keywords:** B-cell non-Hodgkin lymphoma, circulating tumour DNA, liquid biopsy, relapse, droplet digital PCR

## Abstract

Growing evidence now suggests that circulating tumour DNA (ctDNA) has great potential as a non-invasive biomarker for disease monitoring, since ctDNA carries tumour-specific modifications. In particular, monitoring ctDNA has important implications for identifying patients with haematological malignancies at clinical risk of disease progression. We hereby describe three patients with B-cell non-Hodgkin lymphoma and investigate the clinical value of sequential ctDNA profiling for the early detection of tumour relapse. Somatic mutations in diagnostic tumour biopsy samples of these three patients were identified by applying high-throughput next-generation sequencing. Droplet digital PCR probes and primers were designed and tested for each hotspot mutation. Serial ctDNA analysis was subsequently conducted among these three patients. We found that the longitudinal monitoring of plasma ctDNA could predict for at least one month in advance compared with flow cytometry, cytology and conventional imaging modalities. Therefore, our results support liquid biopsy based on ctDNA as a non-invasive complementary modality to other detection methods for detecting early relapse and contribute to more precise management for non-Hodgkin lymphoma patients.

## 1. Introduction

Liquid biopsy, as a non-invasive molecular test, has developed dramatically in recent years, mainly among solid tumours. This non-invasive method can reliably provide a patient’s tumour mutational status by detecting circulating tumour cells (CTCs), circulating tumour DNA (ctDNA), microRNA (miRNA) and extracellular vesicles (EVs) isolated from biological fluids (such as urine and blood samples) [1,2]. To date, liquid biopsy has been extensively used for tracking mutations and monitoring tumour burden and minimal residual disease (MRD) in many malignancies, including non-small-cell lung cancer [3], metastatic melanoma [4], colorectal cancer [5], breast cancer [6,7], and various haematological diseases [8,9].

In the field of lymphoid neoplasms, the value of liquid biopsy has been highlighted in recent literature, demonstrating liquid biopsy as a novel promising tool. Scherer et al. reported that ctDNA genotyping classified transcriptionally defined tumour subtypes, including diffuse large B-cell lymphoma (DLBCL) cell of origin, directly from the plasma [10]. Kurtz et al. demonstrated superior event-free and overall survival in patients who had a 2-log reduction in ctDNA after one cycle of therapy, or a 2.5-log reduction in ctDNA after two cycles of therapy compared to pretreatment levels [11]. Roschewski and colleagues showed that surveillance ctDNA was able to identify the risk of recurrence at a median of 3.5 months before clinical evidence of disease in most DLBCL patients [12]. Taken together, these results suggest that the molecular detection of ctDNA plays a particularly crucial role in early diagnosis, guiding treatment, assessing prognosis, and promoting the personalized management of patients with lymphoma [13].

Herein, we report three cases of B-cell non-Hodgkin lymphoma to investigate the prognostic utility of ctDNA monitoring. Our results indicated that the evaluation of ctDNA levels was able to facilitate the rapid identification of patients at high risk for relapse. Most importantly, this case series of three patients showed that ctDNA levels converted to positive earlier than those obtained from flow cytometry, cytology, and conventional imaging modalities. Overall, our findings suggest that plasma ctDNA can serve as a surrogate biomarker for monitoring early recurrence, and will enable the timely management of patients with non-Hodgkin lymphoma.

## 2. Materials and Methods

### 2.1. Patients

Three patients with relapsed B-cell non-Hodgkin lymphoma were enrolled in this study. Two patients received chimeric antigen receptor-modified (CAR) T cell treatment, and one received chemotherapy. The baseline patient characteristics are detailed in Table S1. This study was approved by the Ethics Committee of Tongji Hospital, Tongji Medical College, Huazhong University of Science and Technology, and all subjects provided written informed consent prior to inclusion in the study in accordance with the TJ-IRB20180813 approved study protocol.

### 2.2. CAR T Cell Production

Third-generation CAR was composed of a single-chain variable fragment from a murine monoclonal antibody against human CD19 or CD22, 2 costimulatory domains from CD28 and 4-1BB, and a CD3ζ signalling domain. The T cells were separated from patients and subsequently stimulated in vitro, as previously described [14]. Lentivirus-mediated CAR transduction was then performed 24 h after culture. Transfection efficiency, apoptosis and tumoricidal activity were assessed to ensure the quality of CAR T cells. The anti-CD22 CAR T and anti-CD19 CAR T cells were cultured in vitro for 14 days, and examinations for viability, mycoplasma, endotoxin and sterility were performed before infusion.

### 2.3. Clinical Protocol for CAR T Cell Therapy

For lymphodepletion chemotherapy, patients received an FC regimen (fludarabine at 25 mg/m^2^ and cyclophosphamide at 20 mg/kg) daily for 3 days (day −4 to day −2) before CAR T-cell infusion, followed by infusion of CAR19 and CAR22 T cells separately in 2 divided doses. The first day of CAR T cell infusion was taken as day 0.

### 2.4. Positron Emission Tomography (PET) Scans

All patients underwent PET/CT with 18F-fluorodeoxyglucose (FDG) after the end of treatment in the nuclear medicine department, and the response was assessed by a clinical evaluation and imaging according to current recommendations [15].

### 2.5. Bone Marrow Cytological Examination

After informed consent was given, bone marrow aspirates were taken under local anaesthesia. Bone marrow smears were subjected to Wright–Giemsa staining according to the standard protocol. Photographic images were acquired for conventional morphological assessment with a Nikon Eclipse 50i microscope, and the original magnifications were all 1000×/numerical aperture 0.65.

### 2.6. Phenotypic Analysis by Flow Cytometry

Bone marrow aspirations, peripheral blood samples and ascites samples were obtained and subjected to flow cytometric analysis. Antibodies against the following proteins were used to identify abnormal cells and the phenotypes: PE-CY7 anti-CD10 (BDIS, San Jose, CA, USA), Pacific Blue anti-CD19 (Biolegend, San Diego, CA, USA), APC-H7 anti-CD20 (BD Pharmingen, San Diego, CA, USA), PE anti-CD22 (BDIS, San Jose, CA, USA), PerCP-Cy5.5 anti-CD38 (BD Pharmingen, San Diego, CA, USA), APC anti-CD79a (Dako, Carpinteria, CA, USA), FITC anti-Igκ (BDIS, San Jose, CA, USA) and PE anti-Igλ (BDIS, San Jose, CA, USA). Data acquisition was conducted on a BD LSRFortessa flow cytometer (BD Biosciences, San Jose, CA, USA).

The detailed gating strategies for flow cytometric analysis were shown as follows. First, cells were gated on forward scatter height (FSC-H) vs. forward scatter area (FSC-A) for single cells and side scatter area (SSC-A) vs. FSC-A for live cells. Next, rough B-cell populations were gated based on SSC-A signals and CD79a expression [16]. The CD79a+ gate was further analysed for the expression of CD10, CD19, CD20 CD22, Igκ and Igλ to detect the malignant cells. Data were processed using Flowjo software (Tree Star Inc., Ashland, OR, USA).

### 2.7. Cell-Free DNA Extraction

Peripheral blood (PB) samples (10 mL) were taken from the patients using EDTA K2 anticoagulation tubes after obtaining informed consent. Cell-free DNA (cfDNA) was extracted from frozen plasma samples by using the QiaAmp Circulating Nucleic Acid Kit (Qiagen) with carrier RNA added before lysis. Then, the extracted cfDNA was stored at -20 °C until droplet digital PCR (ddPCR).

### 2.8. Probe Design

ddPCR assays for detecting *TP53* (wild-type and mutated), *KMT2D* (wild-type and mutated), *B2M* (wild-type and mutated) and *MYD88* (wild-type and mutated) sequences were designed using Primer Express 3.0.1 and labelled with FAM or VIC fluorophores separately (Applied Biosystems, Foster City, CA, USA). The sequences for all primers and probes are listed in Table S2. The false positive rates and thresholds for all ddPCR assays are given in Table S3.

### 2.9. Droplet Digital PCR

ddPCR analysis was performed in a total reaction volume of 20 μL as previously described [17]. Droplets were generated in 8-well cartridges using a QX200 droplet generator (Bio-Rad, Hercules, CA, USA). Droplets were amplified and then read on a two-fluorescence detector (QX200, Bio-Rad). QuantaSoft version 1.7.4 (Bio-Rad) enabled the determination of the mutant copy number and mutant allele fraction of the samples. The gene mutant allele fraction (MAF) was obtained by dividing the gene mutant allele count (MAC) by the gene total allele count (TAC). The MAC was quantified as the number of single-stranded fragments of DNA amplified containing the mutation of interest. The TAC was defined as the sum of the mutant and wild-type copies of the amplified gene, which was measured by total circulating cfDNA. The sequential ctDNA samples were quantitatively tracked. Serum samples were run in triplicate to screen more DNA.

## 3. Case Presentations

### 3.1. Case 1

**Figure 1:**
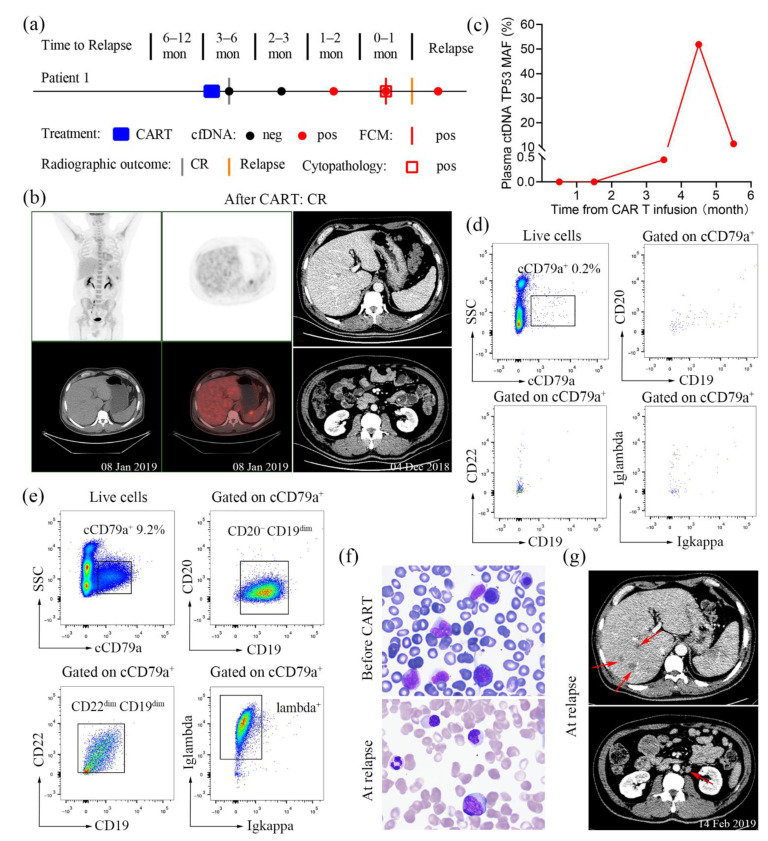
PET/CT image, contrast-enhanced CT image, plasma ctDNA results, and immune and cytological analyses for case 1. (**a**) Summary of patient-level data before relapse. Clinical relapses were confirmed radiographically. Red circle, ctDNA detected; black circle, ctDNA not detected; grey bars, imaging studies demonstrating complete remission; orange bars, imaging studies demonstrating detection of disease; red bars, abnormal cells detected by flow cytometry; red rectangles, naive cells detected. mon, months. (**b**) Fused PET/CT image and contrast-enhanced CT image after CAR T cell therapy, demonstrating complete remission. (**c**) Serial ctDNA monitoring from CAR T cell infusion. (**d**) Phenotypic analysis of peripheral blood cells after CAR T cell therapy on 8 January 2019. (**e**) Phenotypic analysis of the bone marrow aspirate at relapse. (**f**) Wright–Giemsa staining of bone marrow aspirate slides before CAR T cell therapy and at relapse. Original magnifications, 1000×. (**g**) Contrast-enhanced CT image showing new liver lesions and enlarged posterior peritoneum lymph nodes (red arrows) suggestive of tumour relapse.

A 51-year-old male patient was diagnosed with stage IVB DLBCL in February 2018. He received eight cycles of chemotherapy, including two cycles of R-CHOP (rituximab, cyclophosphamide, vincristine, adriamycin and prednisone), three cycles of R-CHOP plus lenalidomide, one cycle of R-CHOEP (R-CHOP plus etoposide), and two cycles of R-DHAP (rituximab, dexamethasone, aracytine and cisplatin). In addition to systemic chemotherapy, intrathecal prophylaxis was delivered with 50 mg cytarabine plus 10 mg methotrexate, plus 5 mg dexamethasone. However, the disease still progressed. Therefore, CAR T cell therapy with anti-CD19 and anti-CD22 CAR constructs was introduced to the patient (Figure 1a). He subsequently achieved complete remission (CR) according to the PET/CT scans (Figure 1b, left panel) and contrast-enhanced CT (Figure 1b, right panel).

On 11 February 2019 (4 months after CAR T cell infusion), he was admitted to our hospital again for consolidation. Plasma ctDNA analysis was performed on the day of admission by using targeted deep sequencing and ddPCR, which revealed the *TP53* p.Cys238Trp mutation with a MAF of 51.88% (Figure 1c). For this patient, plasma ctDNA remained consistently negative for the *TP53* p.Cys238Trp mutation within 3 months after CAR T cell infusion and converted to positive on 8 January 2019, with an MAF of 0.45% (Figure 1c). No abnormal cells were found by multiparameter flow cytometry on 8 January 2019 (Figure 1d). However, the flow cytometric analysis performed on 12 February 2019, showed that 9.2% of nucleated cells were positive for cCD79a, CD19^dim^, CD22^dim^, and lambda and negative for CD20 (Figure 1e). Bone marrow cytology revealed DLBCL with marrow involvement (Figure 1f). However, we evaluated the bone marrow and found no bone marrow infiltration before the initiation of CAR T cell treatment (Figure 1f). Contrast-enhanced abdominal CT performed on 14 February 2019, revealed new liver lesions and multiple markedly enlarged lymph nodes, including the posterior peritoneum lymph nodes (Figure 1g). Collectively, these findings suggested early relapse, which was confirmed by CT and immune and cytological analyses. Therefore, from 15 February 2019, the patient was started on ICE (ifosfamide, carboplatin, and etoposide) plus ibrutinib (560 mg q.d.) to control tumour progression.

### 3.2. Case 2

**Figure 2:**
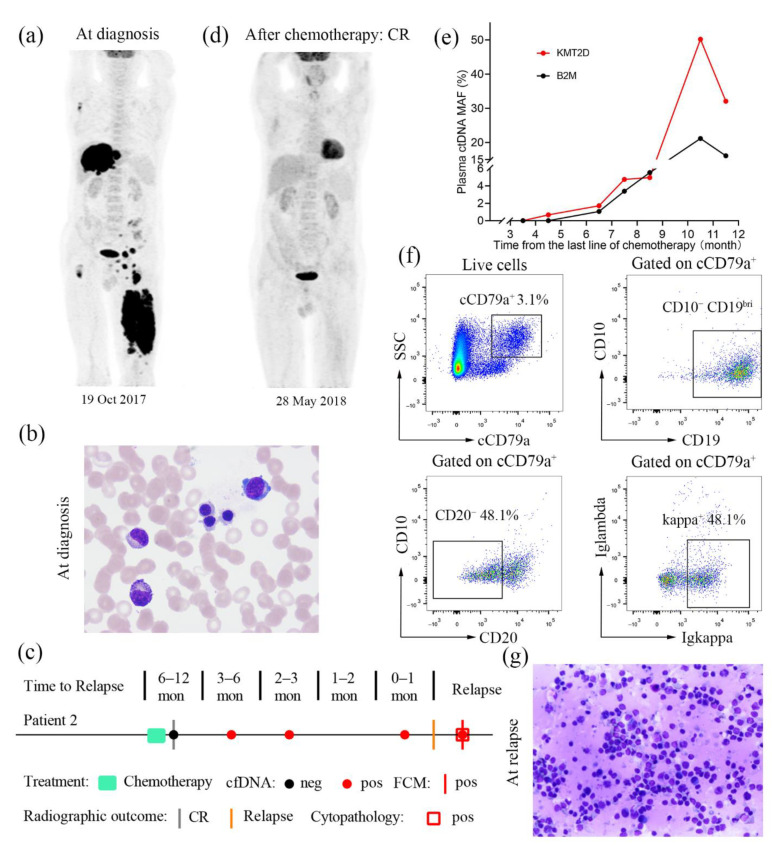
PET/CT image, plasma ctDNA results, and immune and cytological analyses for case 2. (**a**) Whole-body PET/CT imaging at diagnosis (19 October 2017). (**b**) Wright–Giemsa staining of bone marrow aspirate slides at diagnosis. Original magnifications, 1000×. (**c**) Summary of patient-level data before relapse. Clinical relapses were confirmed radiographically. Red circle, ctDNA detected; black circle, ctDNA not detected; grey bars, imaging studies demonstrating complete remission; orange bars, imaging studies demonstrating detection of disease; red bars, abnormal cells detected by flow cytometry; red rectangles, naive cells detected. mon, months. (**d**) Whole-body PET/CT imaging after receiving the last line of chemotherapy (28 May 2018). (**e**) Serial ctDNA monitoring from receiving the last line of chemotherapy. The mutant percentages of KMT2D p.Gln3518* in ctDNA (red) and B2M p.Met1Lys in ctDNA (black) are shown. (**f**) Phenotypic analysis of ascites samples at relapse. (**g**) Wright–Giemsa staining of ascites samples at relapse. Original magnifications, 20×.

A 66-year-old male who was admitted to the Department of Orthopaedic Surgery, Tongji Hospital of Tongji Medical College of Huazhong University of Science and Technology (Wuhan, China) in October 2017 presented with profound swelling and pain on the left thigh. PET/CT was performed on 19 October 2017, and showed highly increased glucose uptake in multiple lesions (Figure 2a). He was pathologically diagnosed with stage IVB DLBCL without bone marrow involvement (Figure 2b) and had an international prognostic index score of 5. The patient was started on induction chemotherapy with six cycles of R-miniCHOP in the Department of Haematology. The patient tolerated the chemotherapy well, with no side effects, and was discharged from the hospital in stable condition. He then received two cycles of single-agent rituximab (Figure 2c) and achieved a PET negative complete remission on 28 May 2018 (Figure 2d).

The patient was admitted to our department again on 11 March 2019, with poor dietary intake and fatigue. Liquid biopsy was performed on his plasma ctDNA and revealed the *KMT2D* p.Gln3518* mutation with a MAF of 50.23%, and the *B2M* p.Met1Lys mutation with an MAF of 21.15% on 12 March 2019 (10 months after chemotherapy), while plasma ctDNA was negative after achieving CR on 28 May 2018, and changed to positive in September 2018 (4 months after chemotherapy), as shown in Figure 2e. Colour Doppler ultrasonography revealed massive abdominal effusions with an unknown aetiology on 22 March 2019, which likely indicated a relapse of DLBCL. On 26 March 2019, flow cytometry of the abdominal effusions revealed 3.1% abnormal cells, 48.1% of which were positive for CD19^bright^, cCD79a^bright^ and kappa and negative for lambda, CD10, CD20 and CD38, and 51.9% of which were positive for CD19^bright^, cCD79a^bright^ and CD20^bright^ and negative for kappa, lambda, CD10 and CD38 (Figure 2f). The patient received a cytological examination of ascites, which showed relapse on 26 March 2019 (Figure 2g). To stop tumour progression, the patient was then given rituximab monotherapy.

### 3.3. Case 3

A 49-year-old female was diagnosed with stage IVA marginal zone lymphoma (MZL) in December 2012. She received a standard induction chemotherapy regimen with R-CHOP for 6 cycles, followed by a complete remission. Then, the patient underwent six courses of rituximab as maintenance therapy between 2014 and 2016. On 18 July 2017, the patient was admitted to a local hospital again, complaining of waist and back pain. Unfortunately, a relapse of MZL was indicated by PET/CT. The patient was treated with immunochemotherapy, using R-DHAP, in 4 cycles of 4 months. A second complete remission (CR2) was achieved after the 2nd cycle of therapy. She continued to receive one cycle of R-CDOP (rituximab, cyclophosphamide, pegylated liposomal doxorubicin, vincristine, and prednisone) in February 2018. After remaining in CR2 for approximately 6 months, the patient experienced relapse in May 2018 and was in a relapsed and refractory status.

**Figure 3:**
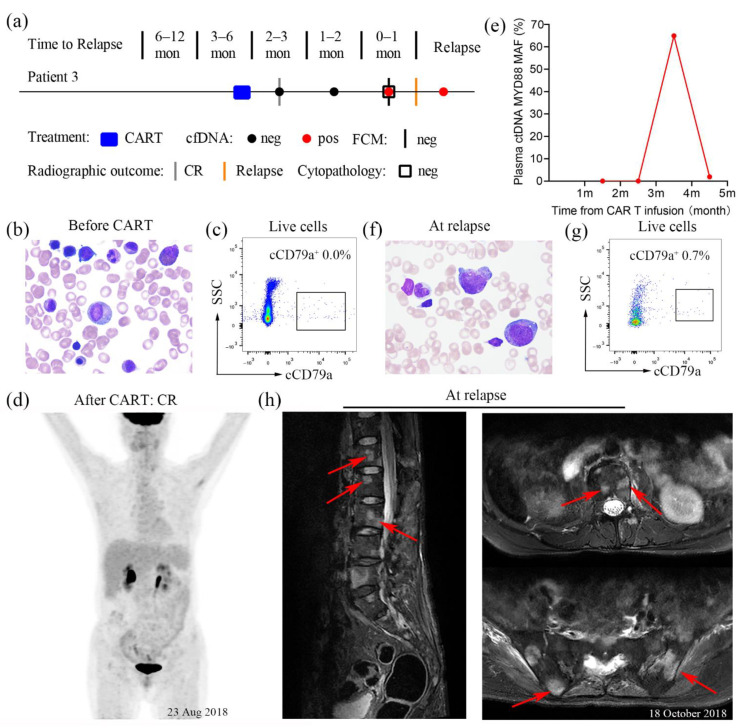
Cytological analysis, flow cytometry analysis, PET/CT image, magnetic resonance image and plasma ctDNA results for case 3. (**a**) Summary of patient-level data before relapse. Clinical relapses were confirmed radiographically. Red circle, ctDNA detected; black circle, ctDNA not detected; grey bars, imaging studies demonstrating complete remission; orange bars, imaging studies demonstrating detection of disease; black bars, abnormal cells not detected by flow cytometry; black rectangles, naive cells not detected; mon, months. (**b**) Wright–Giemsa staining of bone marrow aspirate slides before CAR T cell therapy. Original magnifications, 1000×. (**c**) Phenotypic analysis of peripheral blood cells after CAR T cell therapy on 21 August 2018. (**d**) Whole-body PET/CT image after CAR T cell therapy on 23 August 2018, demonstrating complete remission. (**e**) Serial ctDNA monitoring from CAR T cell infusion. (**f**) Wright–Giemsa staining of bone marrow aspirate slides at relapse. Original magnifications, 1000×. (**g**) Phenotypic analysis of the bone marrow aspirate at relapse. (**h**) Sagittal STIR sequence images and axial T2-weighted images with fat suppression showing multiple abnormal nodular signals in the vertebral body and iliac bones, indicating tumour recurrence; STIR, short time inversion recovery.

To pursue further monitoring and CAR T cell therapy, the patient was admitted to our department on 28 May 2018 (Figure 3a). Bone marrow biopsy on hospital day 2 did not indicate evidence of lymphoma (Figure 3b). High-throughput deep sequencing was performed on hospital day 3, and the *MYD88* p.Leu265Pro mutation was revealed. Subsequently, the patient received a standard lymphodepleting chemotherapy regimen from day −4 to day −2 (16/6/2018 to 18/6/2018). She then received a sequential infusion of curative doses of CD19- and CD22-targeted CAR-T cells. The details were as follows: 4 × 10^6^ cells/kg CAR T 22 on day 0 (20/6/2018), 2 × 10^6^ cells/kg CAR T 22 and 2 × 10^6^ cells/kg CAR T 19 on day + 1 (21/6/2018), followed by 4 × 10^6^ cells/kg CAR T 19 on day + 2 (22/6/2018). After CAR T cell infusions, the patient achieved CR again, as assessed by flow cytometric analysis (Figure 3c) and PET/CT on 23 August 2018 (Figure 3d).

The patient presented to our department for regular screening on 14 October 2018. The *MYD88* p.Leu265Pro mutation was detected on 16 October 2018 (3 months after CAR T cell infusion), with an MAF of 64.95%, which was significantly higher than that obtained on 22 August 2018 (Figure 3e). However, no abnormal cells were observed in the bone marrow aspirate smear (Figure 3f) or flow cytometric analysis (Figure 3g) on 17 October 2018. Subsequently, bone marrow infiltration and MZL relapse were indicated by magnetic resonance imaging on 18 October 2018 (Figure 3h). Consequently, ibrutinib treatment was administered to the patient to control the disease.

## 4. Discussion

Herein, we present three cases which investigate the potential prognostic utility of plasma-based ctDNA monitoring in patients with B-cell non-Hodgkin lymphoma. We identified ctDNA in the baseline samples of these patients, the MAFs of which changed in connection with clinical biomarkers and imaging findings as the disease progressed. Notably, all of the patients exhibited an abundance of ctDNA when early recurrence was observed. Most importantly, the earliest changes in the ctDNA status significantly preceded alterations detected with conventional detection techniques.

Several recent studies have indicated that ctDNA monitoring can be used as a complementary technique to tissue biopsy for tumour mutation analysis in patients with lymphoma [12,18]. Our cases provide favourable evidence in favour of these studies, showing that ctDNA can be used to detect the occult relapse of haematological malignancies. In case 1, an increase in the ctDNA mutation level predicted relapse earlier than that with imaging and flow cytometry analysis, with a one-month lead time (Figure 1a). Likewise, the same results were obtained in case 2, where ctDNA monitoring could better predict relapse 6 months in advance compared with other approaches (Figure 2c). In case 3, flow cytometric analysis for the routine screening of peripheral blood was initially inconclusive; however, ctDNA testing again revealed the *MYD88* p.Leu265Pro mutation consistent with the clinical signs of relapse (Figure 3a). Consistent with our findings, Nakamura et al. indicated that ctDNA-based molecular relapse demonstrated a median 30-day lead time over clinical relapse, and ctDNA monitoring may help identify haematologic cancer patients at risk for relapse in advance of established clinical parameters [19]. As these results collectively show, the liquid biopsy with ctDNA monitoring enables the prediction of disease relapse one to six months earlier than conventional detection methods. Thus, serial ctDNA monitoring in patients with haematological malignancies might be recommended for clinical practice, allowing the early identification of disease relapse and therapy decision-making [12].

The absolutely non-invasive nature of plasma-based liquid biopsies allows repeat testing with a high specificity, capturing dynamic changes in systemic ctDNA levels that can predict relapse after treatment, as demonstrated in cases 1–3. Moreover, ctDNA analysis can provide information about relapse to guide therapeutic strategies and aid in the clinical management of patients with lymphomas. An additional advantage of plasma liquid biopsy is that it overcomes fundamental limitations of tissue biopsy, e.g., sampling biases arising from tumour heterogeneity, the restricted quantity and quality of tumour material available, and the limited number of biopsies [20,21].

However, current clinical ctDNA assays focus on the detection of specific point mutations confirmed by next generation sequencing, often a small panel of genes or several hotspot mutations (mutations in sites in a specific gene known to harbour recurrent alterations that have well-defined biologic and/or clinical significance) [22]. Specifically speaking, *TP53* p.Cys238Trp mutation in case 1, *KMT2D* p.Gln3518* mutation and *B2M* p.Met1Lys mutation in case 2, and *MYD88* p.Leu265Pro mutation in case 3 were found by sequencing and then monitored serially by ddPCR. Thus, this approach provides only a partial glimpse of the tumour genome in plasma because of its targeted nature, and has to be individualized for each patient based on a known landscape of the tumour mutational profile [11]. This disadvantage could be compensated for by ctDNA deep sequencing, which can provide insight into clonogenic evolution and help identify the emergence of new clones.

In conclusion, monitoring circulating tumour DNA plays an important role in identifying non-Hodgkin lymphoma patients at risk of disease recurrence, and has important clinical significance. Unlike other detection assays, testing ctDNA based on ddPCR is a highly specific, non-invasive and dynamic method that can be used as often as necessary to detect subclinical disease. Thus, longitudinal plasma ctDNA profiling can be used as an adjunct to currently available modalities for the management of patients with non-Hodgkin lymphoma.

## Data Availability

The original contributions presented in the study are included in the article/supplementary material. Further inquiries can be directed to the corresponding author.

## Supplementary Materials

The following are available online at https://www.mdpi.com/article/10.3390/diagnostics11112055/s1, Table S1: Clinical data of three patients with B-cell non-Hodgkin lymphoma. Table S2: Sequences of primers used in ddPCR. Table S3: False positive rates and thresholds for all ddPCR assays.
