# Supplementary material for: Longitudinal Monitoring of Plasma Circulating Tumour DNA Enables the Prediction of Early Relapse in Patients with Non-Hodgkin Lymphoma: A Case Series
Source: Diagnostics (Basel). 2021 Nov 5;11(11):2055. doi: 10.3390/diagnostics11112055 (PMC8618087; doi:10.3390/diagnostics11112055)
Supplement: Supplementary file 1 [file diagnostics-11-02055-s001.zip › diagnostics-1418995-supplementary.pdf]

Supplemental Data:

Longitudinal Monitoring of Plasma Circulating Tumour DNA Enables the Prediction of Early Relapse in Patients with Non-Hodgkin Lymphoma: A Case Series

Table S1. Clinical data of three patients with B-cell non-Hodgkin lymphoma.

Case No.	Age at diagnosis (y)	Sex	Disease pathology	Stage	IPI	Gene	Protein change	Primary treatment	Last line of treatment before relapse
1	51	Male	DLBCL non-GCB	IVB	2	*TP53*	p.Cys238Trp	− R-CHOP ×2<br>− R-CHOP+Lenalidomide ×3 (PR)<br>− R-CHOEP ×1<br>− R-DHAP ×2 (PD)	CD22 3.52×10⁶/kg;<br>CD19 3.91×10⁶/kg
2	66	Male	DLBCL non-GCB	IVB	5	*KMT2D*<br>*B2M*	p.Gln3518*<br>p.Met1Lys	− R-miniCHOP ×6	− R ×2
3	49	Female	marginal zone lymphomas	IVA	4	*MYD88*	p.Leu265Pro	− R-CHOP ×6 (CR)<br>− R ×6<br>− R-DHAP ×4 (CR)<br>− R-CDOP ×1 (PD)	CD22 4×10⁶/kg;<br>CD22 2×10⁶/kg,<br>CD19 2×10⁶/kg;<br>CD19 4×10⁶/kg

CR complete remission; DLBCL diffuse large B-cell lymphoma; PD progressive disease; PR partial response; R-CDOP rituximab, cyclophosphamide, pegylated liposomal doxorubicin, vincristine and prednisone; R-CHOP rituximab, cyclophosphamide, vincristine, adriamycin and prednisone; R-CHOEP R-CHOP plus etoposide; R-DHAP rituximab, dexamethasone, aracytine and cisplatin.

Table S2. Sequences of primers used in ddPCR.

Gene	Assay(Protein change)	Nucleotide change	Forward primer	Reverse primer	Mutant probe	WT probe
TP53	p.Cys238Trp	c.714T>G	TCTGACTGTACCACCATCCACTACA	TGGGCCTCCGGTTCATG	CATGTGGAACAGTTCC	CTACATGTGTAACAGTT
KMT2D	p.Gln3518*	c.10552C>T	CTGACCAGCGGCAGTATGAG	CTAGCACCTTCAGCTGCATCTG	CTGTTCCATACCTAGCA	TTCCATACCCAGCAGC
B2M	p.Met1Lys	c.2T>A	GGGCATTCCTGAAGCTGACA	CCTCCAGGCCAGAAAGAGAGA	TTCGGGCCGAGAAGT	TTCGGGCCGAGATGT
MYD88	p.Leu265Pro	c.794T>C	ACTGGGCTTGTCCCACCAT	TCTTTCTTCATTGCCTTGTACTTGA	CATCAGAAGCGACCGA	CATCAGAAGCGACTGA

ddPCR droplet digital PCR, WT wild-type.

Table S3. False positive rates and thresholds for all ddPCR assays.

Assay	False positive rate (%)	Threshold (%)
TP53 p.Cys238Trp	0	0
KMT2D p.Gln3518*	0	0
B2M p.Mel1Lys	0	0
MYD88 p.Leu265Pro	0	0

ddPCR droplet digital PCR.
